# Shared polymorphisms and modifiable behavior factors for myocardial infarction and high cholesterol in a retrospective population study

**DOI:** 10.1097/MD.0000000000007683

**Published:** 2017-09-15

**Authors:** Yulan Liang, Arpad Kelemen

**Affiliations:** a Department of Family and Community Health; b Department of Organizational Systems and Adult Health, University of Maryland, Baltimore, MD.

**Keywords:** association analysis, gene-environment, high cholesterol, myocardial infarction, retrospective population study, single nucleotide polymorphism

## Abstract

Genetic and environmental (behavior, clinical, and demographic) factors are associated with increased risks of both myocardial infarction (MI) and high cholesterol (HC). It is known that HC is major risk factor that may cause MI. However, whether there are common single nucleotide polymorphism (SNPs) associated with both MI and HC is not firmly established, and whether there are modulate and modified effects (interactions of genetic and known environmental factors) on either HC or MI, and whether these joint effects improve the predictions of MI, is understudied.

The purpose of this study is to identify novel shared SNPs and modifiable environmental factors on MI and HC. We assess whether SNPs from a metabolic pathway related to MI may relate to HC; whether there are moderate effects among SNPs, lifestyle (smoke and drinking), HC, and MI after controlling other factors [gender, body mass index (BMI), and hypertension (HTN)]; and evaluate prediction power of the joint and modulate genetic and environmental factors influencing the MI and HC.

This is a retrospective study with residents of Erie and Niagara counties in New York with a history of MI or with no history of MI. The data set includes environmental variables (demographic, clinical, lifestyle). Thirty-one tagSNPs from a metabolic pathway related to MI are genotyped. Generalized linear models (GLMs) with imputation-based analysis are conducted for examining the common effects of tagSNPs and environmental exposures and their interactions on having a history of HC or MI.

MI, BMI, and HTN are significant risk factors for HC. HC shows the strongest effect on risk of MI in addition to HTN; gender and smoking status while drinking status shows protective effect on MI. *rs16944* (gene *IL-1β*) and *rs17222772* (gene *ALOX*) increase the risks of HC, while *rs17231896* (gene *CETP*) has protective effects on HC either with or without the clinical, behavioral, demographic factors with different effect sizes that may indicate the existence of moderate or modifiable effects. Further analysis with the inclusions of gene–gene and gene–environmental interactions shows interactions between *rs17231896* (*CETP*) and *rs17222772* (*ALOX*)*; rs17231896* (*CETP*) and gender. *rs17237890* (*CETP*) and *rs2070744* (*NOS3*) are found to be significantly associated with risks of MI adjusted by both SNPs and environmental factors. After multiple testing adjustments, these effects diminished as expected. In addition, an interaction between drinking and smoking status is significant. Overall, the prediction power in successfully classifying MI status is increased to 80% with inclusions of all significant tagSNPs and environmental factors and their interactions compared with environmental factors only (72%).

Having a history of either HC or MI has significant effects on each other in both directions, in addition to HTN and gender. Genes/SNPs identified from this analysis that are associated with HC may be potentially linked to MI, which could be further examined and validated through haplotype-pairs analysis with appropriate population stratification corrections, and function/pathway regulation analysis to eliminate the limitations of the current analysis.

## 1 Introduction

Risk prediction and assessment are central parts of common complex disease prevention, including coronary heart disease (CHD) and its sequels, such as acute myocardial infarction (MI). Refining prediction strategies remains important for targeting treatment recommendations.^[1–4]^ MI is a leading cause of death throughout the world. Approximately 450,000 people in the United States die from coronary disease per year.^[5]^ The risk of MI increases with age, while the actual incidence is dependent on predisposing risk factors for atherosclerosis.^[6]^ Potential risk factors of MI and atherosclerotic coronary artery disease have been reported as hyperlipidemia, diabetes mellitus, hypertension (HTN), tobacco use, male gender, age, and family history of atherosclerotic arterial disease; genetics.^[7–9]^ Furthermore, family history of heart attacks, lack of physical activity, alcohol consumption, obesity, and stress are also considered as potential associated factors.^[10–13]^ It was noted that having a history of high cholesterol (HC) was associated with a history of MI.^[14–16]^ The genetics on either HC^[17–21]^ or MI^[22,23]^ have been examined. There are a number of studies that reported several of the known MI/CHD loci that are also associated with lipids traits and vice versa.^[24]^ However, whether there are common genotypes or polymorphisms associated with both MI and HC is not confirmed or firmly established,^[25–28]^ which are important and ideal drug or intervention target for precision medicine.^[29–31]^ Furthermore, the genetic variants may play an important, but under-recognized role in modulating the effect of environmental exposures on the risk of MI and HC.^[32–35]^ Currently, whether the joint and modulate effects (including the interactions of both genetic and environmental factors) improve the predictions of MI is understudied. In this article, we will examine whether there are common genetic variants that contribute to the HC and MI from a selected set of genetic variants for retrospective western population studies. Furthermore, we will evaluate the prediction power of these combined effects together with the identified significant environmental factors, which target to test the central hypothesis that a combination of common single nucleotide polymorphisms (SNPs) and environmental factors contributes to the risk of HC as well as MI.

## 2 Methods

### 2.1 Data source, design, study population, and variables

The study sample was randomly selected from the general populations of Erie and Niagara counties in New York State falling into age group 35 to 79 years matched by age within 5-year difference.^[36]^ The study was approved by the ethical committees (institutional review board of the University at Buffalo), and all study participants signed informed consent.^[37]^ For the current analyses, we only used partial data of 1837 Caucasian participants that have been genotyped, of which 818 having history of MI (209 women and 609 men) and 1019 with no history of MI (608 men and 411 women). The data set includes 29 environmental variables collected through participants’ clinic visits, which include health behavior or lifestyle (smoking status, alcohol consumption) in addition to demographic (gender), clinical [body mass index (BMI)], systolic blood pressure (SBP), diastolic blood pressure (DBP), HTN, family history for heart disease, triglycerides (TG), high-density lipoprotein (HDL), low-density lipoprotein (LDL), and total cholesterol. Diabetic patients were excluded from participating in the study.

### 2.2 Genetic marker selection

A number of genes (SNPs) in the metabolic pathways related to MI (i.e., inflammation, inflammation-mediated coagulation, and metalloproteinase activation) from the SeattleSNPs variation discovery panel database (http://pga.gs.washington.edu/) were pre-selected and genotyped on the basis of biological plausibility; significant statistical evidence that SNPs in the gene are consistently associated with variation in intermediate phenotype (e.g., protein levels) or with MI; using linkage disequilibrium and allele frequency (≤10%).^[38–40]^ These genes and SNPs include inflammatory genes Interleukin 1 (*IL-1β*): *rs1143634*, *rs16944*, *rs3917354*, *rs3917356*; and interleukin 6 (*IL 6*): *rs2069825*, *rs1818879*, *rs1548216, rs1800795; CETP* gene*: rs17231513, rs17237890;* Tissue remodeling gene, matrix metalloproteinase (*MMP3*)*: rs522616, rs595840, rs602128, rs680753;* Coagulation gene: tissue factor (*TF*)*: rs1324214, rs1361600, rs3354, rs3917639,* and constitutive and rostane receptor (CAR) gene etc. Genotyping was done in duplicates (success rate 93% to 99%) using the MassARRAY System (Sequenom) in multiplexes with 5 ng of DNA.^[37,40]^ All SNPs are coded as dominant effects [with 1 represents the presence of at least one reference allele (A/a), while 0 is none].

### 2.3 Main outcome measures

MI cases are defined as people who used to have MI (once or multiple) before or during data collection period, and the controls are defined as people who have never had MI by the time of data collection. HC, having a history of HC (once or multiple), are used as a primary lipid outcome measure given some missing issues for other lipid measures for these data.

### 2.4 Data analysis

#### 2.4.1 Imputation-based data analysis for missing data and data preprocessing

Missing data analysis was conducted to examine missing pattern and mechanism, for example, missing of BMI was complete at random (*P* = .178 for little MCAR test). Multiple imputation was performed for near significant variables (*P* < .10), missing ≤10%. These include BMI (2% missing), smoking status (0.3%), HTN (0.1%), Meds for high HTN (6.7%), HC (1.1%), family history for heart disease (8.5%), drinking status in past 1 to 2 years (0.4%), *rs3917356* (*IL-1β1*) (2.2%), *rs2069825* (*IL6*) (2.9%), *ADDU-000614* (*ADDU*) (1.1%), *rs17237890* (*CETP*) (1.7%), *rs2070744* (*NOS*) (2.3%), and *rs2077647* (*ESR1*) (1.9%). There were 3 cases with outliers in amount of ethanol (z = 23.73, 14.33, 11.60), which were deleted, as the amount of ethanol was significantly different by MI. “Drinking status in past 1–2yrs” was recoded to make the sample size in each category less imbalanced: “Lifetime abstainers,” “Irregular abstainers,” and “Non-current drinker” were categorized as “non-current drinker,” “current non-weekly drinker,” “current weekly drinker” as “current drinker.”

#### 2.4.2 Association analysis and predictions

**Figure 1:**
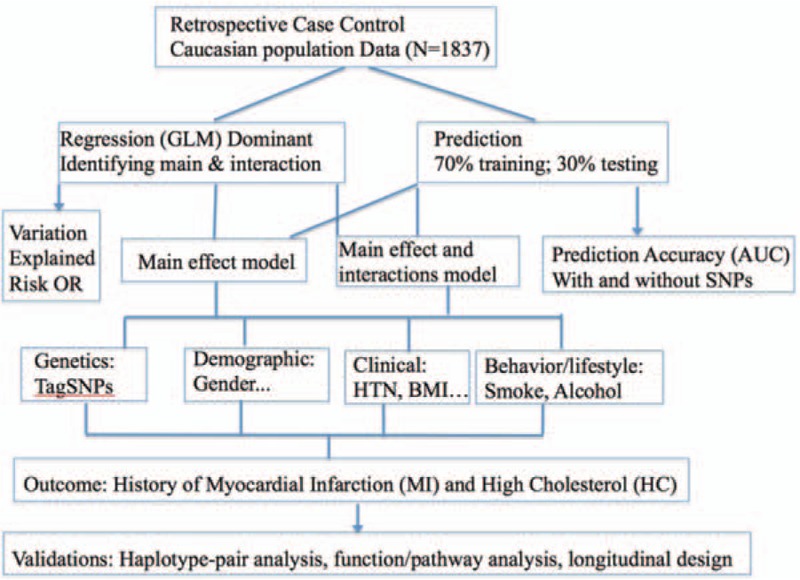
Association and prediction analysis of shared polymorphisms and modifiable environmental (behavior) factors for myocardial infarction and high cholesterol.

The unadjusted univariate association between all factors and MI was calculated and correlation between explanatory variables that may indicate collinearity were assessed pre hoc using Pearson correlation for continuous variables, Chi-square test for categorical variables, and *t* test or analysis of variance (ANOVA) for continuous and categorical variables. Generalized linear models (GLMs) were performed with the dominant genetic effect coding. Besides the genetic factors, demographic, clinical, and lifestyle factors are included as covariates and confounding factors in the multivariable modeling processes, and adjusted odds ratios (ORs) and 95% confidence intervals (95% CIs) were calculated. The model significance was examined using the Omnibus test and the Hosmer and Lemeshow tests. Goodness of fit of model was assessed using deviance/df, AIC, BIC, the model with smaller deviance/df, AIC and BIC values fits the data better. Nagelkerke pseudo *R*^2^ values were reported for the model performance. The presences of outliers (absolute standardized residual values ≥3) that may affect the stability of the model were assessed and removed using plot of leverage value, Cooks distance, and visual inspection. All 2-way interactions between significant explanatory variables were tested and retained in the final model if they significantly improved the model with *P* < .05 based on the *χ*^2^ test of model-fit improvement. For the prediction power of the model, random sampling was conducted to select 70% of the total sample as training data to build up the model, and the other 30% of the sample as testing data; AIC and classification/prediction accuracy are reported (see Fig. 1).

## 3 Results

### 3.1 Sample characteristics

#### 3.1.1 The whole sample

Among all participants, the average age was 54.8 years old [standard deviation (SD) = 8.9]. The majority of the sample was male (66.2%). The average BMI was 28.5 (SD = 5.2), 46.3% had a history of HC, 70.5% had family history for heart disease, and 64% did not have HTN. About two-thirds of the sample drank nonweekly or weekly in the past 1 to 2 years (66.3%), and 43.5% smoked in the past, while 30.6% were current smokers.

#### 3.1.2 MI case–control

In the MI case, 64.9% had a history of HC, while in the non-MI group, only 31.3% had HC, which indicated that HC was associated with MI. These numbers are much higher (due to the age class differences) than the numbers the National Health and Nutrition Examination Survey data reported for 2011 to 2012, which estimated 12.9% of U.S. adults aged 20 years and over (11.1% of men and 14.4% of women; 17.1% of non-Hispanic whites) had HC.^[31–35,41]^ There were significant differences in gender, smoking (lifetime total pack years), drinking status, and amount of ethanol, BMI, SBP, DBP, TG, total cholesterol, HDL, LDL, high blood cholesterol, whether HTN and high blood cholesterol was treated by meds, and family history on heart disease between MI cases and controls (all *P* < .05). Within the MI case group, two-thirds of participants had HC (65.9%), while one-third of participants in the control group had HC (31.6%), which is significantly different (χ^2^ = 210.895, *P* < .001). These descriptive statistics confirm that HC is associated with MI for this Caucasian population. Summary of environmental characteristics for the total sample, the MI cases, and controls is summarized in Table 1.

**Table 1:**
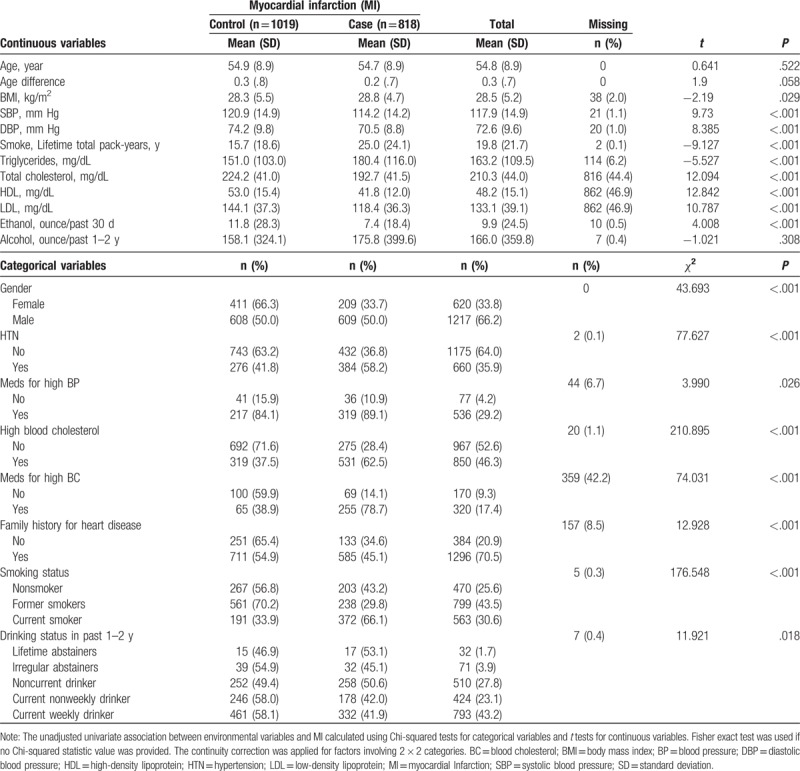
Summary of clinical, behavior, and demographic characteristics and their unadjusted univariate association with MI cases/control status (N = 1837).

### 3.2 Univariate analysis and multivariable for MI

Table 2 provides a summary of genetic characteristics in MI cases and control, as well as the full list of pre-selected and genotyped gene/SNPs. The unadjusted univariate association between tagSNPs and MI calculated using χ^2^ tests are included in Table 2. Risk of MI was associated with SNPs (*P* < .05): *rs3917356* (*IL-1β* gene)*, rs17231513* (*CETP_1*)*, rs17237890* (*CETP_3*), *and rs2070744* (*NOS_2*), while *s2069825* (*IL6*), ADDU-000614 (ADDU), and *rs9340799* (*ESR1*) were near significantly different by MI case and control groups (*P* < .10). None of the rest SNPs was significantly or nearly significantly associated with a risk of MI, which were excluded from multivariable analysis. Finally, 14 genetic and environmental variables (*P* < .10), including BMI, smoking status, HBP, Meds for HTN, HC, family history for heart disease, drinking status in past 1 to 2 years, *rs3917356* (*IL-1β*), *rs2069825* (*il6*), *ADDU-000614* (*ADDU*), *rs17237890* (*CETP*), *rs2070744* (*NOS*), and *rs2077647* (*ESR1*), were included in the follow-up multivariable analysis after multiple imputation.

**Table 2:**
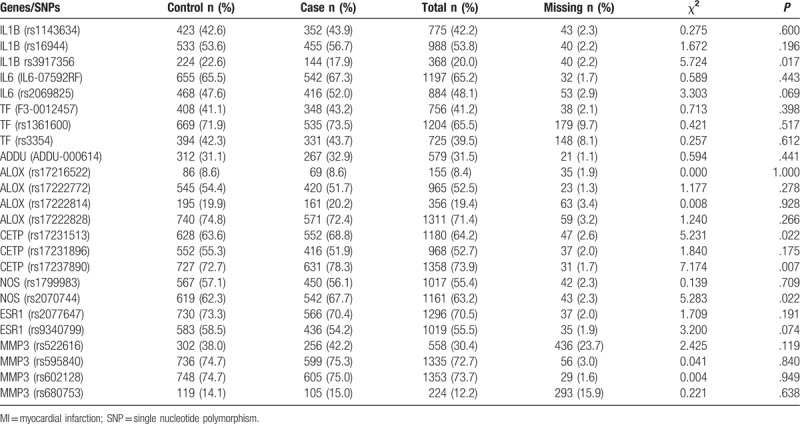
Summary of genetic characteristics in MI cases and control subjects (N = 1837), the unadjusted univariate association between SNPs and MI calculated using Chi-squared tests.

### 3.3 Multivariable analysis for MI

#### 3.3.1 Without SNPs/genes

Age, BMI, gender, HTN, HC, family history about heart disease, high TG, and high alcohol consumption in past 30 days explained 28.5% of the variance (Nagelkerke R squared) in the risk of MI with 72% predictive power for MI status. The strongest factor for MI was the HC [OR: 112.51, 95% CI: 23.10–548.04]. In addition, interactions between HC and age are significant (OR: 0.941, 95% CI: 0.92–0.97).

#### 3.3.2 With both SNPs/genes and the environmental factors with main effects

GLM showed that 8 variables, including gender, smoking status, HTN, HC, family history on heart disease, drinking status in past 1 to 2 years, r*s17231513* (*CETP*), and *rs2070744* (*NOS*) had significant effects in predicting a risk of MI (see Model 1 in Table 3). The model was significant (*P* < .001 in Omnibus Tests, *P* = .671 in Hosmer and Lemeshow Test) and explained 40.6% of total variance in risk of MI (Nagelkerke *R*^2^ = .406), which is higher than without genes/SNPs (28.5%). The prediction power in successfully classifying MI is increased to 78.8% when including both SNPs and environmental factors [area under curve (AUC) = 0.788 (0.751–0.826)] compared with environmental factors (72%).

**Table 3:**
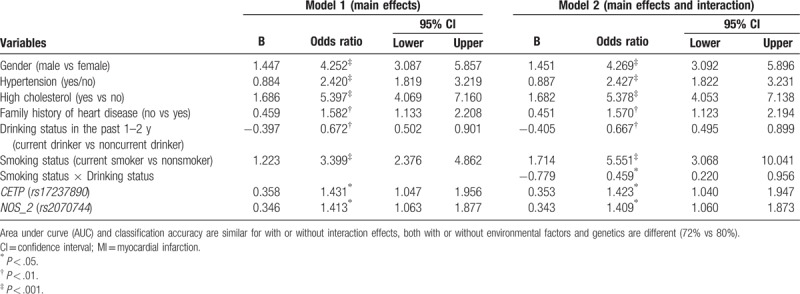
Significant clinical, behavior, demographic, and genetic factors associated with the risk of MI (N = 1834).

HC shows the strongest effect on risk of MI (OR = 5.397, 95% CI 4.069–7.160) compared with other clinical, behavioral, and demographic factors. Smoking status (OR = 3.399, 95% CI 2.376–4.862) for current smoker compared with nonsmoker, HTN (OR = 2.420, 95% CI 1.819–3.219), and gender being male (OR = 4.252, 95% CI 3.087–5.857) are found to be significantly associated with risk of MI. Drinking status in past 1 to 2 years shows protective effect (OR = .672, 95% CI 0.502–0.901). r*s17231513* (*CETP*) (OR = 1.431, 95% CI 1.047–1.956) and *rs2070744* (*NOS*) (OR = 1.413, 95% CI 1.063–1.877) are found to be significantly associated with risk of MI, which are also the most significant SNPs in the unadjusted association analysis. Two other significant SNPs from unadjusted analysis diminish due to the inclusion of conditional or modifiable effects.

#### 3.3.3 With both main effects and interactions of gene and environmental factors

After entering all 2-way interaction effects, the interaction between drinking and smoking status was the only significant effect in the model, which improved the total percent of variance explained by 0.4%. With the interaction effect added, 41% of total variance in risk of MI was explained (Nagelkerke *R*^2^ = 0.410) (see Table 3 Model 2). Similarly, the model fits the data better (Deviance/df = 1.130, AIC/BIC = 691.788/763.237 = 0.906). Overall, the prediction power in successfully classifying MI status is increased to 80% with inclusions of all significant SNPs and environmental factors and their interactions (AUC = 0.799, 0.763–0.836) compared without interactions (78.8%).

HC shows the strongest effect on risk of MI (OR = 5.397, 95% CI 4.069–7.160) compared with other clinical, behavioral, and demographic factors. Smoking status (OR = 3.399, 95% CI 2.376–4.862) for current smokers compared with nonsmokers, HTN (OR = 2.420, 95% CI 1.819–3.219), and being male (OR = 4.252, 95% CI 3.087–5.857) are found to be strongly associated with risk of MI (*P* < .001). Drinking status in the past 1 to 2 years shows protective effect (OR = 0.672, 95% CI 0.502–0.901). Interaction between drinking and smoking status is the only significant protective effect (OR = 0.459, 95% CI 0.220–0.956) for current smokers who drank in the past 1 to 2 years. Two polymorphisms, *rs17237890* (gene *CETP*) (OR = 1.42, 95% CI 1.04–1.95) and *rs2070744* (gene *NOS3*) (OR = 1.41, 95% CI 1.06–1.87), are found to be significantly associated with a risk of MI adjusted by other SNPs and environmental factors.

#### 3.3.4 Univariate and Multivariable analysis for HC

Table 4 provides the univariate analysis and the unadjusted risk using χ^2^ tests, ORs, and 95% CIs for HC for each selected individual genes/SNPs. When adjusted by other genes (SNPs) (see Table 5), with dominant genetic model, the presence of at least one reference allele of *rs16944* (*IL-1β*) increases the risk of HC by 27% (OR = 1.27, 95% CI 1.05–1.55), and *rs17222772* (*ALOX*) increases the odds of the likelihood of HC (OR = 1.42, 95% CI 1.03–1.97), respectively. The presence of at least one reference allele, of SNP *rs17231896* (*CETP*), reduces the risk by 21% (OR = 0.79, 95% CI: 0.65–0.96). While adjusting for the clinical, behavioral, and demographic factors (see Table 6), the risk of HC for *rs17231896* (*CETP*) is 33% lower (OR = 0.67, 95% CI: 0.51–0.88). The increase of effect size (from 21% to 33%) without or with the inclusion of the environmental factors may indicate modifiable effects exist. Table 7 provides the adjusted ORs and 95% CIs for HC predicted by significant genetic, demographic, behavioral, and clinical factors, with both main and interactions effects included. Both BMI and HTN are significant risk factors for HC. With the inclusions of gene–gene and gene–environmental interactions, the multiplicative effects between *rs17231896* (*CETP*) and *rs17222772* (*Alox*); *rs17231896* (*CETP*) and gender; MI and age; *rs9340799* (*ESR1*) and family history of heart diseases were found significantly associated with HC. As expected, after multiple testing (e.g., Bonferroni or false discover rate) adjustments, the significant SNPs for MI or HC diminished to be statistically nonsignificant.

**Table 4:**
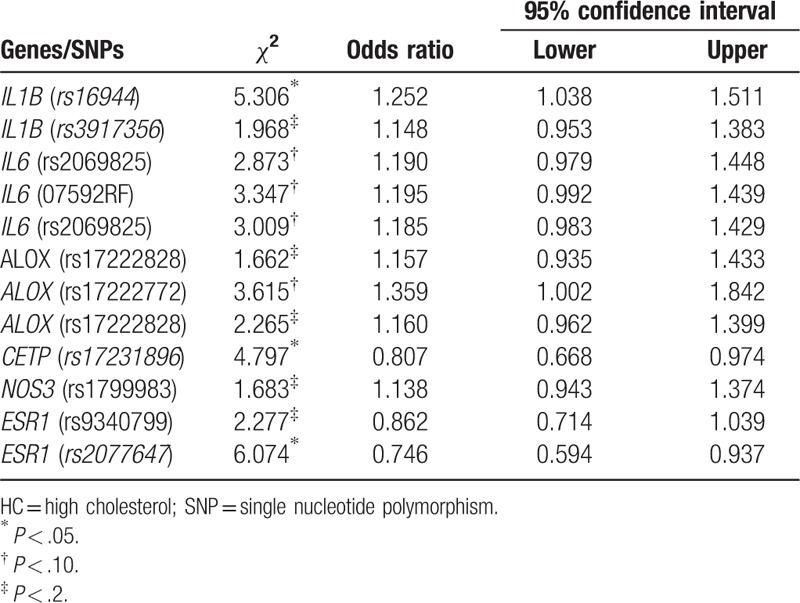
Unadjusted odds ratios and 95% confidence intervals for HC by genes/SNPs (N = 1837).

**Table 5:**
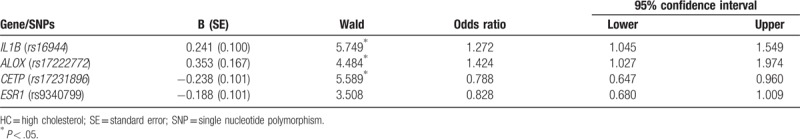
Adjusted odds ratios and 95% confidence intervals for HC predicted by SNPs/genes only adjusted by other gene/SNPs (N = 1837).

**Table 6:**
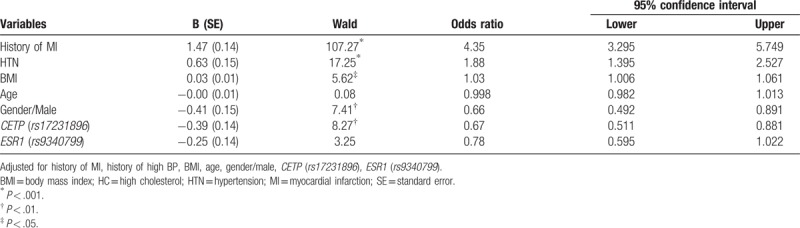
Adjusted odds ratios and 95% confidence intervals for HC predicted by genetic, demographic, behavior, and clinical factors with main effects (N = 1837).

**Table 7:**
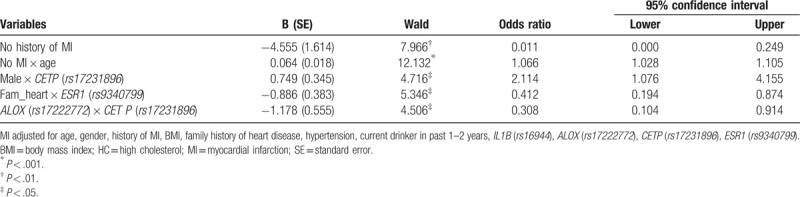
Adjusted odds ratios and 95% confidence intervals for HC predicted by genetic, demographic, behavior, and clinical factors, with main and interactions effects included (N = 1837).

## 4 Discussion

Having a history of either HC or MI has a significant effect on each other in both directions. Common environmental (e.g., smoke, HTN, gender) and genetic factors (gene *CETP*) for both HC and MI are identified from separate analysis with or without adjustment of other significant factors. The association between *CETP* polymorphism (*rs17231896*, *rs17237890,* and *rs17231513*) has protective effects on HC, which is consistent and replicated with the meta-analysis study from ARIC data using genome-wide association study for the association between *CETP* and HDL, in which 3 SNPs from *CETP, rs708272,* (OR = 0.95), *rs5882* (OR = 0.94), and *rs1800775* (OR = 0.95) have been found to be significantly associated with HDL adjusted by both gene and environmental interactions.^[32]^ From a functional content point of view, *CETP* is known to regulate the process of transporting cholesterol from the peripheral arteries to the liver, which helps reduce the risk of CHD. Other studies have also found that CETP expression is regulated by multiple functional SNPs, affecting splicing and transcription, with increased or decreased CETP function, although they focus on selected different variants (*rs247616*, *rs173539*).^[42–44]^

The modulations and joint effects among genes, clinical, behavioral, and other factors on HC and MI are complex. A study from 1995 demonstrated how the *CETP* gene regulating above process is influenced (positively) by alcohol, but subsequent studies have not been able to fully replicate the result.^[45]^ Our analysis confirms that alcohol and smoking habits have effects on MI when both genetic and other factors are adjusted. With inclusions of genes and their interactions, these joint and modulating genetic and lifestyle, clinical factors improve the prediction power for MI and HC. Moreover, both gene–gene and gene–environmental interaction effects were found to be significantly associated with MI and HC, which could be further examined through pathway/regulations and function analysis.^[46]^

It is known that appropriate multiplicity adjustment (either false discovery rate or Bonferroni procedure) is crucial to guarantee the replicability and reproducibility of findings, which should be conducted in large-scale genome-wide association analysis to avoid the potential false positives for multi-trait association analysis.^[47]^ Despite the protective effect of *CETP* on HC, *rs17237890* (different *SNP* of *CETP*) has shown the increased risk on MI when other significant lifestyle factors were included such as smoking and alcohol assumptions. Future haplotype-pair analysis (to reduce the number of tests and increase the expected effect using the haplotypes instead of single genotypes) could be conducted for further validating plausibly gene (i.e., *CETP*) without using all the gene set (for better power) to see whether consistently associated with all the studied phenotypes.^[40,48]^ In addition, within populations of European Caucasian decent, population stratification issue will be assessed and weighted using genomic control with the inflation factor to avoid the spurious association findings.^[49,50]^

The common alleles/genes associated with both MI and HC discovered include *rs16944* (*IL-1β*) with an independent effect without other genetic or environmental factors. However, when adding others, some effects either diminished to statistically nonsignificant or effect size modified, which may indicate some under-recognized role of modulate and modifiable effect of environmental exposures for the genes on the risk of both conditions. The diminished effects (OR with or close to zero) or other potentially missing heritable components of disease etiology may be better further fully explored either through epigenetics analysis^[46]^ with longitudinal data or more complex network and pathway-based analysis, for example, using Bayesian networks for analyzing the direct and indirect probabilistic causal associations to dissect the complex relationships among the significant factors (age, gender, BMI, HTN, smoking, alcohol drinking status, *CETP* gene, CHD/MI).

One limitation of this study is the notable missing data for lipid measures, such as total cholesterol, HDL, LDL, and TG (missing ranged from 44.4% to 46.9%), which is why having a history of HC (once or multiple) is chosen as the primary lipid outcome measure in this paper. Many published works regarding lipid–SNP associations use those measures as primary outcome. Furthermore, for HC treated by meds (statin treatment or other cholesterol-lowering therapies) variable, only 26.6% of participants reported presence or absence such medication, which does not make it feasible to include it in the estimation model. Cases of familial dyslipidemia are not collected for this study and these related lipid variables may change estimated effects and should be addressed and taken into account in future studies.

## References

[1] Wray NR Goddard ME Visscher PM Prediction of individual genetic risk to disease from genome-wide association studies. Genome Res 2007;17:1520–8. 17785532 10.1101/gr.6665407 PMC1987352

[2] Smith JA Ware EB Middha P Current applications of genetic risk scores to cardiovascular outcomes and subclinical phenotypes. Curr Epidemiol Rep 2015;2:180–90. 26269782 10.1007/s40471-015-0046-4 PMC4527979

[3] Paynter NP Chasman DI Paré G Association between a literature-based genetic risk score and cardiovascular events in women. JAMA 2010; 303:631–637. 20159871 10.1001/jama.2010.119 PMC2845522

[4] Holm H Thorleifsson G Stefansson K Genetic risk score and cardiovascular events in women. JAMA 2010;303:2032–3. 20501922 10.1001/jama.2010.660

[5] Mozaffarian D Benjamin EJ Go AS Heart disease and stroke statistics--2015 update: a report from the American Heart Association. Circulation 2015;131:e29–322. 25520374 10.1161/CIR.0000000000000152

[6] Canto JG Rogers WJ Goldberg RJ NRMI Investigators. Association of age and sex with myocardial infarction symptom presentation and in-hospital mortality. J Am Med Assoc 2012;307:813–22. 10.1001/jama.2012.199 PMC4494682 22357832

[7] Yusuf S Hawken S Ounpuu S INTERHEART Study Investigators. Effect of potentially modifiable risk factors associated with myocardial infarction in 52 countries (the INTERHEART study): case-control study. Lancet 2004;364:937–52. 15364185 10.1016/S0140-6736(04)17018-9

[8] Merry AH Boer JM Schouten LJ Smoking, alcohol consumption, physical activity, and family history and the risks of acute myocardial infarction and unstable angina pectoris: a prospective cohort study. BMC Cardiovasc Disord 2011;11:13. 21435252 10.1186/1471-2261-11-13 PMC3073941

[9] Kessler T Erdmann J Schunkert H Genetics of coronary artery disease and myocardial infarction. Curr Cardiol Rep 2013;15:368. 23616109 10.1007/s11886-013-0368-0

[10] Heidemann C Hoffmann K Klipstein-Grobusch K Potentially modifiable classic risk factors and their impact on incident myocardial infarction: results from the EPIC-potsdam study. Eur J Cardiovasc Prev Rehab 2007;14:65–71. 10.1097/01.hjr.0000238392.19847.4c 17301629

[11] Kathiresan S Srivastava D Genetics of human cardiovascular disease. Cell 2012;148:1242–57. 22424232 10.1016/j.cell.2012.03.001 PMC3319439

[12] Abdulla J Kober L Abildstrom SZ Impact of obesity as a mortality predictor in high-risk patients with myocardial infarction or chronic heart failure: a pooled analysis of five registries. Eur Heart J 2008;29:594–601. 18270214 10.1093/eurheartj/ehn010

[13] Labounty TM Gomez MJ Achenbach S Body mass index and the prevalence, severity, and risk of coronary artery disease: an international multicentre study of 13 874 patients. Eur Heart J Cardiovasc Imaging 2013;14:456–63. 22922955 10.1093/ehjci/jes179 PMC3708721

[14] Lindquist P Bengtsson C Lissner L Cholesterol and triglyceride concentration as risk factors for myocardial infarction and death in women, with special reference to influence of age. J Intern Med 2002;251:484–9. 12028503 10.1046/j.1365-2796.2002.00985.x

[15] Heart Protection Study Collaborative Group. MRC/BHF Heart Protection Study of cholesterol lowering with simvastatin in 20,536 high-risk individuals: a randomised placebo-controlled trial. Lancet 2002;360:7–22.12114036

[16] Li JX Cao J Lu XF The effect of total cholesterol on myocardial infarction in Chinese male hypertension population. Biomed Environ Sci 2010;23:37–41. 20486434 10.1016/S0895-3988(10)60029-3

[17] Kim DS Burt AA Ranchalis JE Novel gene-by-environment interactions: APOB and NPC1L1 variants affect the relationship between dietary and total plasma cholesterol. J Lipid Res 2013;54:1512–20. 23482652 10.1194/jlr.P035238 PMC3622343

[18] Kathiresan S Melander O Guiducci C Six new loci associated with blood low-density lipoprotein cholesterol, high-density lipoprotein cholesterol or triglycerides in humans. Nat Genet 2008;40:189–97. 18193044 10.1038/ng.75 PMC2682493

[19] Kathiresan S Melander O Anevski D Polymorphisms associated with cholesterol and risk of cardiovascular events. N Engl J Med 2008;358:1240–9. 18354102 10.1056/NEJMoa0706728

[20] Kathiresan S Willer CJ Peloso GM Common variants at 30 loci contribute to polygenic dyslipidemia. Nat Genet 2009;41:56–65. 19060906 10.1038/ng.291 PMC2881676

[21] Sandhu MS Waterworth DM Debenham SL LDL-cholesterol concentrations: a genome-wide association study. Lancet 2008;371:483–549. 18262040 10.1016/S0140-6736(08)60208-1 PMC2292820

[22] Matsuoka R Abe S Tokoro F Association of six genetic variants with myocardial infarction. Int J Mol Med 2015;35:1451–9. 25738804 10.3892/ijmm.2015.2115

[23] Wallace C Newhouse SJ Braund P Genome-wide association study identifies genes for biomarkers of cardiovascular disease: serum urate and dyslipidemia. Am J Hum Genet 2008;82:139–49. 18179892 10.1016/j.ajhg.2007.11.001 PMC2253977

[24] Keenan TE Rader DJ Genetics of lipid traits and relationship to coronary artery disease. Curr Cardiol Rep 2013;15:396. 23881580 10.1007/s11886-013-0396-9 PMC3883430

[25] Keavney B Palmer A Parish S International Studies of Infarct Survival (ISIS) Collaborators. Lipid-related genes and myocardial infarction in 4685 cases and 3460 controls: discrepancies between genotype, blood lipid concentrations, and coronary disease risk. Int J Epidemiol 2004;33:1002–13. 15256516 10.1093/ije/dyh275

[26] Morgan TM Krumholz HM Lifton RP Nonvalidation of reported genetic risk factors for acute coronary syndrome in a large-scale replication study. JAMA 2007;297:1551–61. 17426274 10.1001/jama.297.14.1551

[27] Cohen JC Boerwinkle E Mosley TH Sequence variations in PCSK9, low LDL, and protection against coronary heart disease. N Engl J Med 2006;354:1264–72. 16554528 10.1056/NEJMoa054013

[28] Willer CJ Sanna S Jackson AU Newly identified loci that influence lipid concentrations and risk of coronary artery disease. Nat Genet 2008;40:161–9. (2007). 18193043 10.1038/ng.76 PMC5206900

[29] Holmes M Keating B Asselbergs F Mendelian randomization of blood lipids for coronary heart disease. Eur Heart J 2015;36:539–50. 24474739 10.1093/eurheartj/eht571 PMC4344957

[30] Brown G Albers JJ Fisher LD Regression of coronary artery disease as a result of intensive lipid-lowering therapy in men with high levels of apolipoprotein B. N Engl J Med 1990;323:1289–98. 2215615 10.1056/NEJM199011083231901

[31] Ha V Sievenpiper J de Souza RJ Effect of dietary pulse intake on established therapeutic lipid targets for cardiovascular risk reduction: a systematic review and meta-analysis of randomized controlled trials. CMAJ 2014;186:E252–62. 24710915 10.1503/cmaj.131727 PMC4016088

[32] Brautbar A Ballantyne CM Lawson K Impact of adding a single allele in the 9p21 locus to traditional risk factors on reclassification of coronary heart disease risk and implications for lipid-modifying therapy in the Atherosclerosis Risk in Communities (ARIC) study. Circ Cardiovasc Genet 2009;2:279–85. 20031596 10.1161/CIRCGENETICS.108.817338 PMC2771929

[33] Teslovich TM Musunuru K Smith AV Biological, clinical and population relevance of 95 loci for blood lipids. Nature 2010;466:707–13. 20686565 10.1038/nature09270 PMC3039276

[34] Mäkelä KM Seppälä I Hernesniemi JA Genome-wide association study pinpoints a new functional apolipoprotein B variant influencing oxidized low-density lipoprotein levels but not cardiovascular events: AtheroRemo Consortium. Circ Cardiovasc Genet 2013;6:73–81. 23247145 10.1161/CIRCGENETICS.112.964965

[35] Elbers CC Guo Y Tragante V Gene-centric meta-analysis of lipid traits in African, East Asian and Hispanic populations. PLoS One 2012;7:e50198. 23236364 10.1371/journal.pone.0050198 PMC3517599

[36] Clarke A Donahue RP Dorn J Franco OH Cross-cultural comparison of correlates of quality of life and health status: the Whitehall II Study (UK) and the Western New York Health Study (US). Eur J Epidemiol 2012;27:255–65. 22392587 10.1007/s10654-012-9664-z PMC3370162

[37] McCann SE Sempos C Freudenheim JL Alcoholic beverage preference and characteristics of drinkers and nondrinkers in western New York (United States). Nutr Metab Cardiovasc Dis 2003;13:2–11. 12772432 10.1016/s0939-4753(03)80162-x

[38] Bis JC Heckbert SR Smith NL Variation in inflammation-related genes and risk of incident non- fatal myocardial infarction or ischemic stroke. Atherosclerosis 2008;198:166–73. 17981284 10.1016/j.atherosclerosis.2007.09.031 PMC2517173

[39] Gigante B Bennet AM Leander K The interaction between coagulation factor 2 receptor and interleukin 6 haplotypes increases the risk of myocardial infarction in men. PLoS One 2010;5:e11300. 20585578 10.1371/journal.pone.0011300 PMC2891999

[40] Gaetano M Quacquaruccio G Di Castelnuovo A Haplotypes and haplotype-pairs of IL-1 beta and IL-6 genes and risk of non fatal myocardial infarction in the Western New York Acute MI Study. Thromb Haemost 2011;106:1231–3. 22072054 10.1160/TH11-06-0377

[41] Roger VL Go AS Lloyd-Jones DM American Heart Association Statistics Committee and Stroke Statistics Subcommittee. Heart disease and stroke statistics--2012 update: a report from the American heart association. Circulation 2012;125:e2–20. 22179539 10.1161/CIR.0b013e31823ac046 PMC4440543

[42] Suhy A Hartmann K Papp A Regulation of cholesteryl ester transfer protein expression by upstream polymorphisms: reduced expression associated with rs247616. Pharmacogenet Genomics 2015;25:394–401. 26061659 10.1097/FPC.0000000000000151 PMC4499003

[43] Suhy A Hartmann K Newman L Genetic variants affecting alternative splicing of human cholesteryl ester transfer protein. Biochem Biophys Res Commun 2014;443:1270–4. 24393849 10.1016/j.bbrc.2013.12.127 PMC3929938

[44] Papp AC Pinsonneault JK Wang D Transfer protein (CETP) polymorphisms affect mRNA splicing, HDL levels, and sex-dependent cardiovascular risk. PLos One 2012;7:e31930. 22403620 10.1371/journal.pone.0031930 PMC3293889

[45] Tolstrup JS Gronbaek M Nordestgaard BG Alcohol intake, myocardial infarction, biochemical risk factors, and alcohol dehydrogenase genotypes. Circ Cardiovasc Genet 2009;2:507–14. 20031627 10.1161/CIRCGENETICS.109.873604

[46] Zanoni P Khetarpal SA Larach DB CHD Exome+ Consortium; CARDIoGRAM Exome Consortium; Global Lipids Genetics Consortium. Rare variant in scavenger receptor BI raises HDL cholesterol and increases risk of coronary heart disease. Science 2016;351:1166–71. 26965621 10.1126/science.aad3517 PMC4889017

[47] Peterson C Bogomolov M Benjamini Y Many phenotypes without many false discoveries: error controlling strategies for multitrait association studies. Genet Epidemiol 2016;40:45–56. 26626037 10.1002/gepi.21942 PMC4738479

[48] De Gaetano M Donati MB Trevisan M Tissue factor gene polymorphisms and haplotypes and the risk of ischemic vascular events: four studies and a meta-analysis. J Thromb Haemost 2009;7:1465–71. 19583819 10.1111/j.1538-7836.2009.03541.x

[49] Campbell CD Ogburn EL Lunetta KL Demonstrating stratification in a European American population. Nat Genet 2005;37:868–72. 16041375 10.1038/ng1607

[50] Price AL Butler J Patterson N Discerning the ancestry of European Americans in genetic association studies. PLoS Genet 2008;4: 10.1371/journal.pgen.0030236 PMC2211542 18208327

